# Multimodality Imaging of Thoracic Outlet Syndrome: Etiological and Anatomical Correlates

**DOI:** 10.3390/diagnostics16101437

**Published:** 2026-05-08

**Authors:** Çağlar Uzun, Sena Ünal, Ebru Düşünceli Atman, Elif Peker, Ayşegül Gürsoy Çoruh, Namik Kemal Altinbas, Ayten Kayi Cangir

**Affiliations:** 1 Department of Radiology, Faculty of Medicine, Ankara University, 06230 Ankara, Turkey; drsenaunal@gmail.com (S.Ü.); ebrumd2001@yahoo.com (E.D.A.); elifozyurek0@yahoo.com (E.P.); draysegulgursoy@gmail.com (A.G.Ç.); namikaltin@gmail.com (N.K.A.); 2 Department of Thoracic Surgery, Faculty of Medicine, Ankara University, 06230 Ankara, Turkey; kayicangir@gmail.com

**Keywords:** thoracic outlet syndrome (TOS), CT, MRI, US, DSA

## Abstract

Thoracic outlet syndrome (TOS) is a rare neurovascular compression disorder involving the brachial plexus and/or subclavian vessels at the cervicothoracobrachial junction. Clinical presentation is often nonspecific and may overlap with peripheral neuropathies, cervical spine disorders, and musculoskeletal conditions, making diagnosis challenging. Although clinical evaluation remains central to diagnosis, imaging plays a complementary role in supporting the diagnosis of TOS by identifying the affected neurovascular structures, localizing the site of compression, and elucidating the underlying anatomical or pathological causes. Moreover, imaging is essential for excluding alternative diagnoses, including thoracic malignancies and inflammatory or degenerative vascular diseases that may mimic TOS. This review provides a comprehensive overview of the radiological anatomy of the thoracic outlet, the etiological factors contributing to neurovascular compression, and the strengths and limitations of current imaging modalities used in the evaluation of TOS.

## 1. Introduction

Thoracic outlet syndrome (TOS) is a neurovascular compression disorder that occurs at the thoracic outlet, also referred to as the cervicothoracobrachial junction, and may be precipitated by certain postural maneuvers. The diagnosis is primarily clinical, based on patient-reported symptoms, physical examination findings, and provocative maneuvers. Electrophysiological studies are mainly useful for excluding alternative diagnoses and may provide supportive findings in selected cases but are not required for establishing a diagnosis, particularly in neurogenic TOS. Imaging plays a complementary role by identifying the affected neurovascular structures, localizing the site and extent of compression, and demonstrating underlying anatomical or pathological causes. In addition, imaging is important for detecting thoracic malignancies—particularly lung cancer—that may invade the chest wall and present with symptoms mimicking TOS [1,2,3]. This review aims to provide a comprehensive overview of the imaging modalities and radiological findings relevant to the diagnosis of TOS, with particular emphasis on the anatomical, etiological, and clinical considerations pertinent to its evaluation.

## 2. Thoracic Outlet Syndrome

TOS is classified into three subtypes according to the compressed structure: arterial TOS (involving the subclavian artery), venous TOS (involving the subclavian vein), and neurogenic TOS (involving the brachial plexus). In some cases, multiple types of compression may occur concurrently. Compression of the subclavian artery may result in ischemic neuritis of the brachial plexus, presenting with pain, weakness, and a sensation of coldness in the affected limb. Subclavian vein compression typically manifests as pain, swelling, and a feeling of heaviness due to venous stasis. Neurogenic TOS, caused by compression of the brachial plexus, presents with symptoms such as pain, dysesthesia, numbness, and weakness [4]. Repetitive arterial compression may lead to vascular changes, including stenosis, poststenotic aneurysmal dilatation, thrombosis, and distal embolization in some cases of arterial TOS [5]. In venous TOS, axillosubclavian vein thrombosis may develop—a condition first described by Paget in 1875 and von Schroetter in 1884, and now collectively referred to as Paget–Schroetter syndrome [6,7]. Although neurogenic and arterial TOS may occur simultaneously due to their anatomical proximity within the same compartment, venous TOS most commonly presents as an isolated entity. Nevertheless, venous and neurogenic TOS may coexist in approximately 5–10% of cases [8].

Thoracic outlet syndrome (TOS) is a relatively rare condition, and its true prevalence cannot be precisely determined. Although the estimates reported in the literature have limited validity, the incidence has been suggested to range between 0.3% and 8% [9]. It exhibits a marked female predominance, with a female-to-male ratio of approximately 4:1, and most commonly affects individuals between 20 and 40 years of age [10]. Neurogenic TOS constitutes the majority of cases, accounting for more than 95% of diagnoses. In contrast, vascular forms of TOS are considerably less common, with venous TOS representing 2–3% and arterial TOS less than 1% of cases [11]. Despite its recognized clinical importance, the true incidence and prevalence of TOS may be underestimated due to variability in diagnostic criteria and inconsistent reporting practices across studies. To promote standardization, the Society for Vascular Surgery has established reporting guidelines for patients with TOS, and the American College of Radiology (ACR) has issued recommendations regarding the appropriate use of imaging modalities in its diagnosis [8,10]. Notably, TOS predominantly affects individuals during their most productive years. When left undiagnosed or inadequately managed, the condition may lead to significant functional impairment, work disability, and reduced socioeconomic productivity, thereby amplifying its broader public health and societal impact [2].

## 3. Radiological Anatomy

The thoracic outlet extends medially to the cervical spine and mediastinum, and laterally to the inferior border of the pectoralis minor muscle. Anatomically, it is divided into three distinct compartments: the interscalene triangle, the costoclavicular triangle, and the retropectoralis minor space [1].

**Figure 1:**
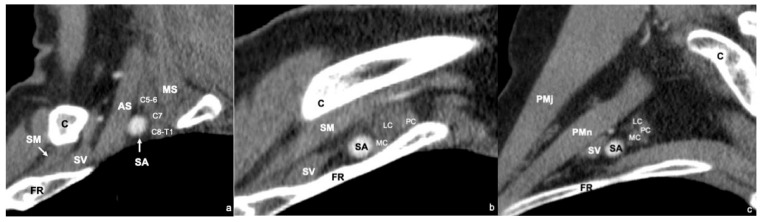
Sagittal reformatted CT images demonstrate the boundaries of the interscalene triangle (**a**), costoclavicular space (**b**), and retropectoralis minor space (**c**), along with the anatomical structures contained within them. C = clavicle; FR = first rib; AS = anterior scalene muscle; MS = middle scalene muscle; SM = subclavius muscle; SA = subclavian artery; SV = subclavian vein; C5 = fifth cervical nerve root; C6 = sixth cervical nerve root; C7 = seventh cervical nerve root; C8 = eighth cervical nerve root; T1 = first thoracic nerve root; PC = posterior cord; LC = lateral cord; MC = medial cord; PMj = pectoralis major muscle; PMn = pectoralis minor muscle.

The interscalene triangle is the most medial compartment. It is bounded anteriorly by the anterior scalene muscle, posteriorly by the middle and posterior scalene muscles, and inferiorly by the first rib. This space contains the subclavian artery and three trunks of the brachial plexus. The subclavian artery lies at the base and anterior aspect of the triangle, whereas the brachial plexus trunks are positioned posteriorly. From superior to inferior, these include the superior trunk (C5–C6), middle trunk (C7), and inferior trunk (C8–T1), all in close proximity to the artery. The subclavian vein courses anterior to the anterior scalene muscle and does not pass through the interscalene triangle [1] (Figure 1a).

The costoclavicular space constitutes the middle compartment of the thoracic outlet. It is bordered superiorly by the clavicle, anteriorly by the subclavius muscle, inferiorly by the first rib, and posteriorly by the anterior scalene muscle. Within this compartment, the subclavian vein lies anteriorly, the subclavian artery is located immediately posterior to it, and the three cords of the brachial plexus occupy the most posterior and superior position. The lateral cord is formed by anterior divisions of the superior and middle trunks, the medial cord by the anterior division of the inferior trunk, and the posterior cord by posterior divisions of all three trunks [1] (Figure 1b).

The most lateral compartment is the retropectoralis minor space. It is bounded anteriorly by the pectoralis minor muscle, posteriorly and superiorly by the subscapularis muscle, and posteriorly and inferiorly by the chest wall. This space contains the axillary artery and vein, along with the cords of the brachial plexus. The spatial relationship of the vascular and neural structures in this compartment closely resembles that observed in the costoclavicular space [1] (Figure 1c).

The anatomy of the thoracic outlet is dynamic, and compartmental narrowing may be induced by postural maneuvers, particularly during arm elevation. CT and MRI studies in asymptomatic individuals have demonstrated that elevation of the upper extremity does not significantly affect the dimensions of the interscalene triangle but results in narrowing of both the costoclavicular space and the retropectoralis minor space [12,13,14]. In neurogenic TOS, compression most commonly occurs within the interscalene triangle and the retropectoralis minor space. Venous compression predominantly involves the costoclavicular space and, less frequently, the retropectoralis minor space. Arterial compression is most often observed in the interscalene triangle and the costoclavicular space [8,13]. According to the Society for Vascular Surgery guidelines, neurogenic and venous compressions occurring within the retropectoralis minor space should be classified as *neurogenic pectoralis minor syndrome* and *venous pectoralis minor syndrome*, respectively. To date, an arterial pectoralis minor syndrome has not been defined [8].

## 4. Etiology of TOS

Both congenital and acquired abnormalities in the bony structures and soft tissues that form and constrain the thoracic outlet can lead to compression of neurovascular structures. Additionally, certain postural and morphotype characteristics may predispose individuals to thoracic outlet syndrome [1] (Table 1).

### 4.1. Bone Abnormalities

**Figure 2:**
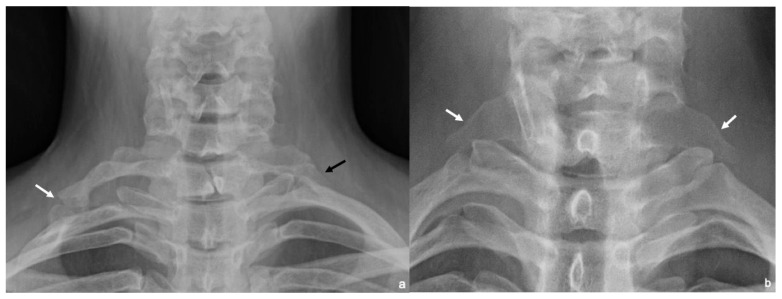
(**a**) Cervical plain radiograph shows a complete cervical rib on the right articulating with the first rib (white arrow) and an incomplete cervical rib on the left without articulation (black arrow). (**b**) Bilateral elongated C7 transverse processes extend beyond and slightly inferior to the T1 transverse processes (white arrows). T1 = first thoracic vertebra; C7 = seventh cervical vertebra.

Cervical rib is an additional rib that arises from the seventh cervical vertebra (C7), most often detected incidentally on routine chest radiographs. Its prevalence is estimated at 0.2–1% in the general population, and it is observed in 5–9% of patients with TOS [1,2,15]. Cervical ribs are asymptomatic in most patients and do not require resection. First described by Gruber in 1869, cervical ribs are classified into four types: (1) cervical ribs extending just beyond the transverse process, (2) cervical ribs extending beyond the transverse process with a free tip almost touching the first rib, (3) cervical ribs extending beyond the transverse process with fibrous bands or cartilage attaching to the first rib, and (4) cervical ribs completely fused to the first rib. The latter two types are more likely to cause complications such as arterial compression, thrombosis, or aneurysm formation [15]. An alternative classification system divides cervical ribs into complete and incomplete forms. Incomplete cervical ribs do not articulate directly with the first rib but are often connected via a fibrous band. In contrast, complete cervical ribs articulate with the first rib either through a joint or by bony fusion. Articulation typically occurs via a tubercle near the insertion of the anterior scalene muscle, a region where the interscalene triangle narrows, displacing the subclavian artery anteriorly and increasing the risk of vascular compression [1,2] (Figure 2a).

An elongated C7 transverse process is defined as the extension of the C7 transverse process beyond and slightly inferior to the transverse process of the first thoracal (T1) vertebra (Figure 2b). Although more common than the cervical rib, the majority of cases are asymptomatic. The incidence in the general population is reported to be as high as 18–23%, with population studies indicating a higher prevalence among patients with TOS [16]. It is distinguished from the cervical rib by the absence of a costovertebral joint. An elongated transverse process can cause compression by itself or through an accompanying fibrous band or an abnormal middle scalene muscle [2].

**Figure 3:**
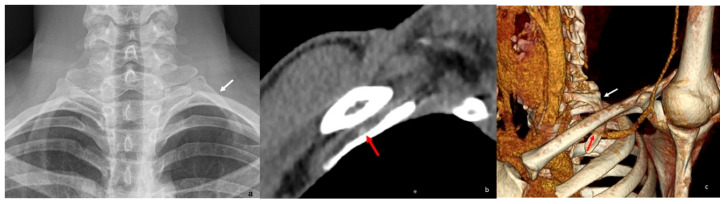
(**a**) Cervical plain radiograph demonstrates a hypoplastic first rib on the left (white arrow). (**b**) Sagittal reformatted CT image of the same patient shows compression of the subclavian artery between the clavicle and the hypoplastic first rib (red arrow), with marked narrowing of the costoclavicular space. (**c**) 3D reformatted images more clearly depict the relationship between the compressed subclavian artery (red arrow) and the hypoplastic first rib (white arrow) within the costoclavicular space. 3D = three-dimensional.

Congenital anomalies of the first rib—such as a broad first rib or an enlarged scalene tubercle—can contribute to narrowing of the thoracic outlet [17]. A hypoplastic first rib that articulates with the second rib instead of the sternum may also lead to neurovascular compression, often through associated fibrous bands or a hypertrophic joint [18] (Figure 3). Acquired conditions, including exostoses, tumors, hypertrophic callus formation or nonunion resulting from previous fractures of the first rib or clavicle, are additional potential causes of TOS [19,20,21,22,23,24].

### 4.2. Soft Tissue Abnormalities

**Figure 4:**
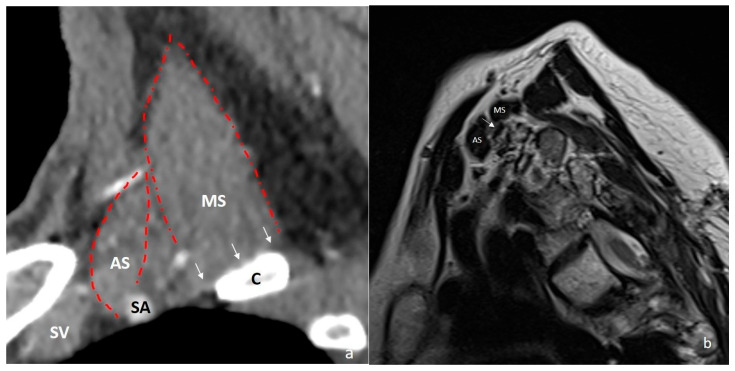
(**a**) Sagittal reformatted CT image demonstrates a hypertrophic middle scalene muscle, which narrows the interscalene triangle due to its broad insertion on the clavicle (white arrows). The red dashed lines indicate the boundaries of the anterior and middle scalene muscles. (**b**) Scalenus minimus muscle: on sagittal T1-weighted MRI, muscle fibers originating from the middle scalene muscle and coursing between the brachial plexus elements are observed (white arrow). C = clavicle; AS = anterior scalene muscle; MS = middle scalene muscle; SA = subclavian artery; SV = subclavian vein.

Several congenital soft tissue anatomical variations may further compromise the thoracic outlet. These include fibrous bands; hypertrophy or abnormal insertions of the anterior scalene muscle; distal division of a single-origin anterior and middle scalene muscle; the brachial plexus passing between fibers of the scalene muscles; interdigitation of scalene muscles; insertion of the middle scalene muscle onto the first rib with a broad base; and the presence of accessory muscles such as the scalenus minimus (Figure 4). These variations can reduce the dimensions of the interscalene triangle and the costoclavicular space, contributing to TOS [1,17]. Hypertrophy of the subclavius muscle can similarly narrow the costoclavicular space. Additionally, the presence of an accessory subclavius posticus muscle—extending from the anterior portion of the first rib to the upper medial border of the scapula—has been implicated in TOS due to its proximity to the brachial plexus and subclavian vessels [25,26]. In neurogenic TOS, a hyperactive pectoralis minor muscle is known to cause dynamic compression of the brachial plexus. Over time, chronic muscle contraction and fibrosis can lead to progressive narrowing of the retropectoralis minor space [27,28].

**Figure 5:**
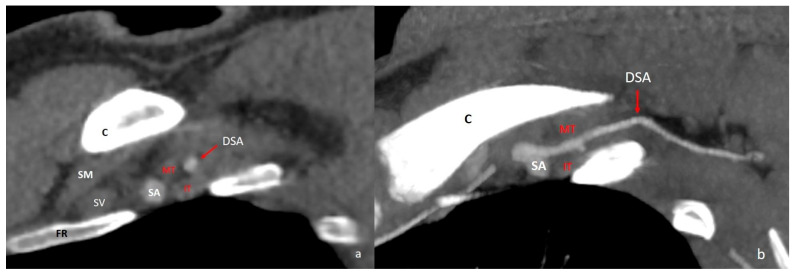
Dorsal scapular artery arising from the second part of the subclavian artery. Sagittal reformatted (**a**) and oblique MIP CT images (**b**) demonstrate its course between the middle and inferior trunks of the brachial plexus. C = clavicle; FR = first rib; SM = subclavius muscle; SA = subclavian artery; SV = subclavian vein; DSA = dorsal scapular artery; MT = middle trunk; IT = inferior trunk; MIP = maximum intensity projection.

The dorsal scapular artery accompanies the dorsal scapular nerve and supplies the levator scapulae and rhomboid muscles. Variations in its origin are clinically important, as they can contribute to neurogenic TOS due to the artery’s anatomical relationship with the brachial plexus [29,30]. The artery most commonly originates from the transverse cervical artery (48%). It arises less frequently from the third part (25%) and the second part (22%) of the subclavian artery, and only rarely from the axillary artery (5%) [31]. When the dorsal scapular artery originates from the transverse cervical artery, it rarely passes through the brachial plexus (3%). However, in cases where it arises from the subclavian artery, it passes between the fibers of the brachial plexus in 100% of cases from the second part and in 75% of cases from the third part. When it originates from the subclavian artery, it passes between the upper and middle trunks in 40% of cases and between the middle and lower trunks in 23% of cases [29] (Figure 5).

Posttraumatic and postoperative fibrous scarring can also contribute to compression at the thoracic outlet. In cases of flexion–extension trauma to the neck, both the surrounding muscles and fibers of the brachial plexus may sustain injury, potentially resulting in the formation of fibrotic scars. Repetitive microtraumas—often seen in sports, occupational tasks, and other activities that involve frequent overhead arm movements or heavy lifting—can lead to fibrosis and spasms in the scalene and subclavius muscles. These changes may elevate the first rib and compress adjacent neurovascular structures [17].

### 4.3. Predisposing Postural or Morphologic Factors

In droopy shoulder syndrome, as well as in thin women with poor posture and weak shoulder muscles, there is an increased risk of TOS due to narrowing of the acromioclavicular and costoclavicular spaces [32].

### 4.4. Pathologies That Mimic TOS Symptoms

**Figure 6:**
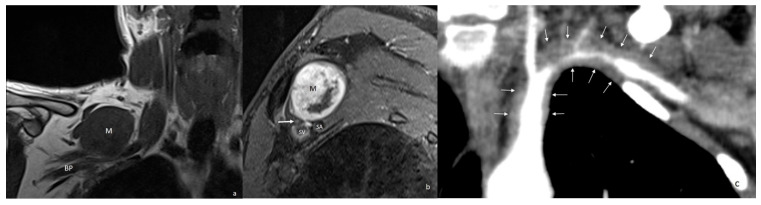
In a patient presenting with symptoms mimicking TOS, coronal T1-weighted (**a**) and sagittal post-contrast T1-weighted (**b**) MR images demonstrate a well-circumscribed, heterogeneously enhancing mass (schwannoma) compressing the brachial plexus (white arrow). (**c**) In another patient with TOS-like symptoms diagnosed with Takayasu arteritis, the arterial-phase coronal reformatted MIP CT image shows diffuse concentric wall thickening of the left subclavian artery (white arrows) with severe stenosis in its mid-segment. BP = brachial plexus; M = mass; SA = subclavian artery; SV = subclavian vein; MIP = maximum intensity projection.

In addition to diagnosing conditions that directly cause compression at the thoracic outlet, it is essential to exclude pathologies that may mimic TOS symptoms. These include superior sulcus (Pancoast) tumors of the lung that invade the thoracic outlet, lipomas and neurogenic tumors in the supraclavicular region, and large-vessel vasculitis—particularly Takayasu arteritis—which can cause thickening and stenosis of the subclavian and axillary arteries (Figure 6). Significant arterial stenosis due to advanced atherosclerosis may also present with symptoms similar to TOS [2,33,34,35].

## 5. Imaging Techniques

### 5.1. Plain Radiography

Cervical spine and chest radiographs should be obtained during the initial evaluation to assess bony structures. These imaging studies can help identify anatomical variations and abnormalities such as cervical ribs, an elongated C7 transverse process, and congenital or acquired deformities of the first rib and clavicle, as well as focal bone lesions. Although the negative predictive value of chest radiographs is low for detecting small tumors, they can still be useful in identifying large thoracic masses [10,27].

### 5.2. Computed Tomography and Computed Tomographic Angiography

Because CT protocols for TOS differ from standard examinations in both positioning and acquisition parameters, the suspected diagnosis must be explicitly stated in the imaging request [27]. The examination is performed in two positions: with the arms in a neutral position and in hyperabduction. For computed tomographic angiography (CTA), intravenous contrast administration is required [10,12]. To optimize visualization of the subclavian and axillary arteries and avoid venous streak artifacts, the contrast should be injected into the arm opposite the side being examined. A total of 90 mL of iodinated contrast is administered via the antecubital vein at a rate of 4 mL/s. Scanning typically begins 15–20 s after injection, or timing can be adjusted using bolus tracking. During imaging, the symptomatic arm is first scanned in a neutral (adducted) position, while the contralateral arm is abducted to reduce streak artifacts. Then, the symptomatic arm is placed in abduction while the opposite arm is adducted to minimize artifacts. If venous TOS is clinically suspected, additional delayed imaging may be performed at approximately 90 s post-injection [1,2,10,12].

Reconstruction using thin-slice thickness (1–2 mm), two-dimensional (2D) reformatted images and three-dimensional (3D) volumetric images is recommended. Studies have shown that the degree of stenosis can be misjudged when assessed using axial images alone [10]. In one study, stenosis was incorrectly measured in 43% of cases using only axial images, and in 10% of cases using only sagittal reformatted images. Therefore, sagittal reformatted images derived from axial data should be included in the evaluation to more accurately determine the location and severity of arterial compression [36].

**Figure 7:**
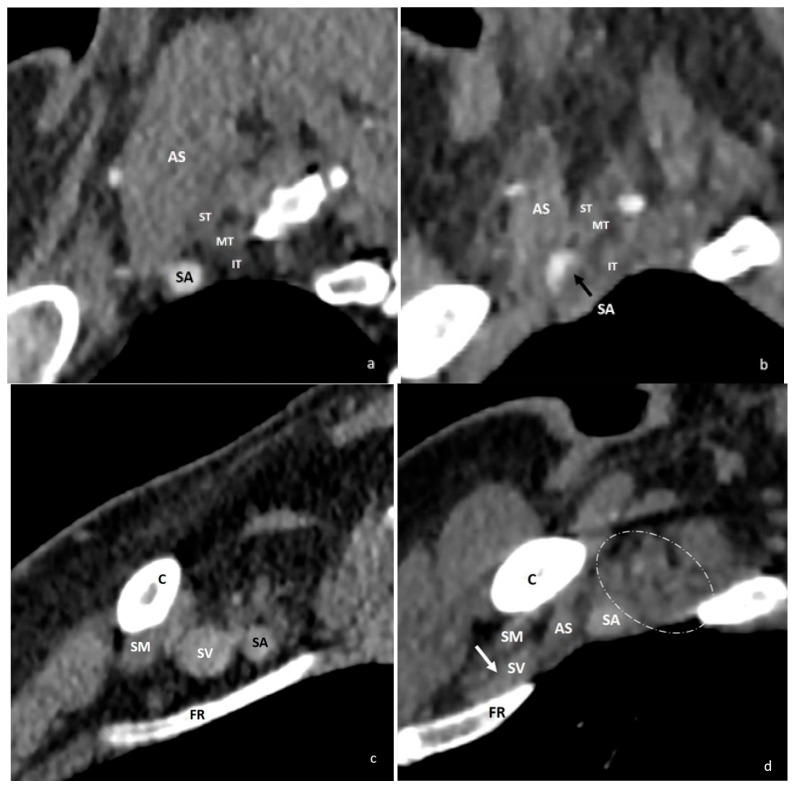
At the level of the interscalene triangle, arterial-phase sagittal reformatted CT images show that the subclavian artery (**a**), which has a normal caliber in the neutral position, becomes compressed by the anterior scalene tendon during arm hyperabduction (**b**) (black arrow). At the level of the costoclavicular space, the subclavian vein (**c**), normal in caliber in the neutral position, is compressed between the subclavius muscle and the clavicle during hyperabduction (**d**) (white arrow). Narrowing of the costoclavicular space with hyperabduction results in crowding of the brachial plexus elements (circled area). C = clavicle; FR = first rib; AS = anterior scalene muscle; SA = subclavian artery; SV = subclavian vein; SM = subclavius muscle; ST = superior trunk; MT = middle trunk; IT = inferior trunk.

The degree of stenosis is assessed by comparing the decrease in cross-sectional diameter or area of the vessel between the neutral and postural maneuver positions [1,10,12]. A decrease of more than 30% in the subclavian artery diameter and more than 50% in the subclavian vein diameter between these positions has been considered diagnostic of TOS [10,37] (Figure 7). However, it is important to note that positional vascular compression can occur in both symptomatic and asymptomatic individuals. For instance, studies have shown that positional compression of the subclavian vein occurs in approximately 52% of asymptomatic individuals, and subclavian artery compression occurs in about 11% [13,37]. CTA findings of arterial compression have demonstrated good correlation with intraoperative findings [10,38]. Despite this, diagnosing venous TOS remains more challenging due to the higher prevalence of asymptomatic compression. Therefore, the diagnosis of vascular TOS should not rely solely on imaging evidence of positional vessel narrowing. Clinical correlation is essential, as diagnosing TOS based only on vascular compression may lead to misdiagnosis and potentially unnecessary surgical interventions [1,10,13]. A recent study comparing CTA and magnetic resonance angiography (MRA) found that CTA demonstrated superior intraobserver and interobserver agreement in measuring arterial and venous stenosis [39].

**Figure 8:**
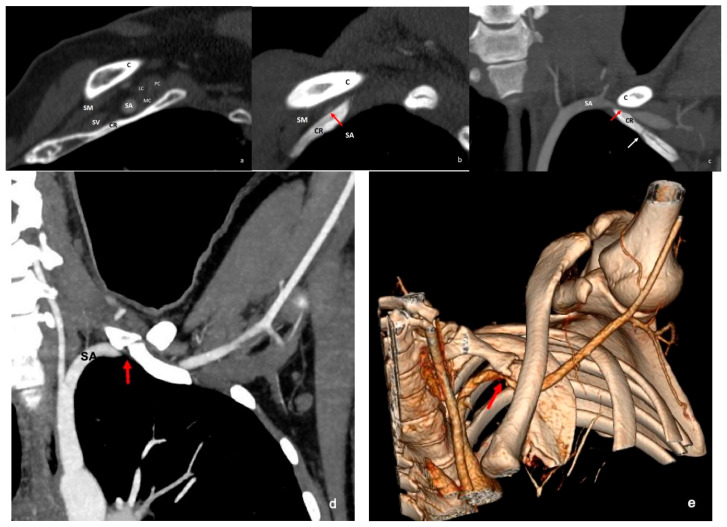
In the neutral position, arterial-phase sagittal reformatted CT shows a subclavian artery of normal caliber within the costoclavicular space (**a**). With arm hyperabduction, marked compression of the subclavian artery is demonstrated on oblique reformatted CT (**b**) and coronal MIP images (**c**) (red arrows). The coronal MIP image also demonstrates a complete cervical rib articulating with the first rib (white arrow). In another patient, coronal MIP arterial-phase CT (**d**) and 3D CT (**e**) images show a complete cervical rib articulating with the first rib and compression of the subclavian artery at this level (red arrows). C = clavicle; CR = cervical rib; SM = subclavius muscle; SA = subclavian artery; SV = subclavian vein; LC = lateral cord; MC = medial cord; PC = posterior cord; MIP = maximum intensity projection; 3D = three-dimensional.

**Figure 9:**
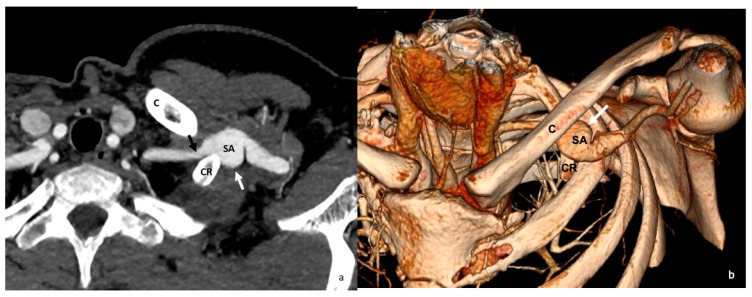
Arterial-phase axial CT (**a**) and 3D CT (**b**) images obtained with the arm in hyperabduction demonstrate compression of the subclavian artery at the level of the complete cervical rib (black arrow) and a poststenotic aneurysm of the subclavian artery (white arrows). C = clavicle; CR = cervical rib; SA = subclavian artery; 3D = three-dimensional.

**Figure 10:**
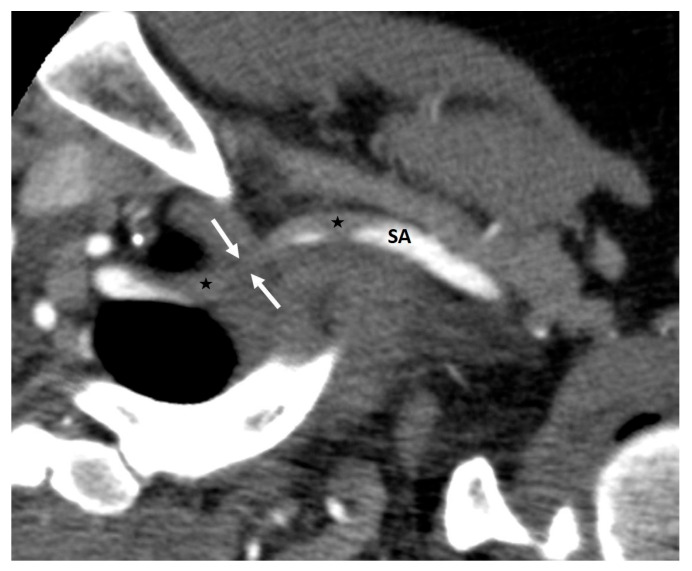
Arterial-phase axial CT obtained during arm hyperabduction demonstrates compression of the subclavian artery within the costoclavicular space (white arrows), with hypodense intraluminal filling defects consistent with thrombus both proximal and distal to the site of compression (black stars). SA = subclavian artery.

The addition of 2D reformatted images and 3D volumetric reconstructions enhances the visualization of the anatomical relationships between bony structures and vascular elements (Figure 8). This approach also improves detection of poststenotic dilatation (Figure 9). Thrombosis, which can be seen with or without poststenotic dilatation in arterial TOS, can also be successfully visualized with CTA (Figure 10). While venous compression alone is less predictive of TOS, CTA is valuable for identifying more definitive, late-stage findings such as venous thrombosis and collateral circulation [1,10]. The CTA protocol for TOS is summarized in Table 2.

Due to the limited contrast resolution for soft tissues, CT has a restricted role in the diagnosis of neurogenic TOS [2]. However, CT is superior to plain radiography in evaluating bone anatomy and detecting bony abnormalities. It is particularly effective in identifying superior sulcus pathologies that may contribute to TOS symptoms [10]. Additionally, CT enables the quantification of dynamic changes in the costoclavicular space and interscalene triangle during provocative maneuvers, providing further diagnostic insight [12].

### 5.3. Magnetic Resonance Imaging—Magnetic Resonance Angiography

**Figure 11:**
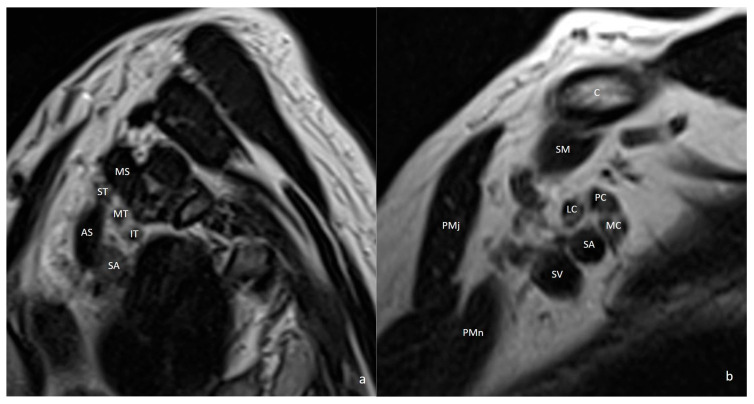
T2-weighted sagittal MR images demonstrate the normal anatomy of the brachial plexus, adjacent muscles, and vascular structures (**a**,**b**). C = clavicle; AS = anterior scalene muscle; MS = middle scalene muscle; SA = subclavian artery; SV = subclavian vein; SM = subclavius muscle; ST = superior trunk; MT = middle trunk; IT = inferior trunk; PC = posterior cord; LC = lateral cord; MC = medial cord; PMj = pectoralis major muscle; PMn = pectoralis minor muscle.

Since clinical findings often overlap with peripheral neuropathies, cervical radiculopathy, and musculoskeletal injuries, the diagnosis of neurogenic TOS can be challenging [2,40]. Magnetic resonance imaging (MRI) is the modality of choice for evaluating the brachial plexus, adjacent musculature, and vascular structures owing to its excellent soft tissue contrast and multiplanar capabilities, all without ionizing radiation (Figure 11). MRI provides not only an accurate anatomical assessment but also depiction of pathological changes within the nerve fibers such as edema, inflammation, and degeneration. In addition, muscle abnormalities such as edema, denervation, and fatty atrophy can also be visualized [41].

In brachial plexus imaging, both 1.5 Tesla (T) and 3T scanners can be used. 3T systems offer a higher signal-to-noise ratio (SNR) and contrast-to-noise ratio (CNR), enabling higher spatial resolution and improved visualization of fine nerve structures [42,43]. However, they are more prone to susceptibility artifacts. Due to more pronounced artifacts at 3T, their use may not be suitable for all patients. In particular, in patients with metallic foreign bodies or implanted orthopedic instruments, 1.5 T scanners are generally preferred. Optimal imaging requires careful coil configuration, typically involving a combination of head-neck, body, and surface coils to maximize signal reception [41]. In the imaging of brachial plexus fibers, both 2D and 3D sequences should be used. While 2D imaging provides better spatial resolution, 3D imaging allows for multiplanar reconstruction, enabling a more accurate assessment of the course of the nerves. In addition to axial plane images, coronal and oblique sagittal planes targeted to the brachial plexus should also be acquired [42].

Axial and coronal T1-weighted images (WI) without fat suppression allow for clear delineation of the nerves due to the surrounding fat tissue, and also enable the evaluation of fatty muscle atrophy. Axial and coronal fat-suppressed T2-WI or STIR (short tau inversion recovery) images are useful in assessing pathological signal changes in the nerves as well as edema in the muscles. Oblique sagittal images without fat suppression, obtained perpendicular to the brachial plexus, facilitate the evaluation of the relationship of nerve roots, trunks, divisions, cords, and branches with adjacent structures. Additional oblique sagittal T2-WI may be obtained while performing the provocative maneuver. Although the routine use of contrast-enhanced imaging remains controversial, in patients with a suspected mass, fat-suppressed contrast-enhanced T1-weighted axial, coronal, and oblique sagittal images may be obtained [41].

On MRI, normal nerves appear isointense to muscle on T1-WI and isointense or slightly hyperintense relative to muscle on T2-WI. Due to the longitudinal orientation of the collagen fibers within the nerves, a magic angle artifact may occur and on STIR sequences, nerve fibers may appear hyperintense. To avoid misinterpretation as a pathological signal increase, the nerve fibers should always be compared with those on the contralateral side [42].

**Figure 12:**
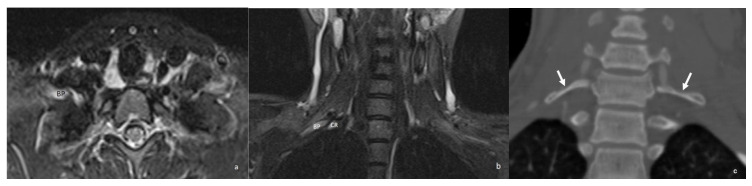
Axial (**a**) and coronal (**b**) STIR MR images demonstrate hyperintensity of the brachial plexus consistent with edema and a cervical rib causing compression. (**c**) A coronal reformatted CT image of the same patient demonstrates bilateral cervical ribs (white arrows). STIR = short tau inversion recovery; BP = brachial plexus; CR = cervical rib.

Mild T2 hyperintensity of the C8 and T1 nerve roots is a common and often insignificant finding. However, when this abnormal T2 signal extends into the lower trunk and the nerve becomes enlarged, it usually aligns with the clinical symptoms. Pathologies originating from the interscalene triangle and the minor pectoral region may cause neurogenic TOS. MRI can demonstrate compression, fibrous bands, muscle abnormalities, or accessory muscles. Compression of the brachial plexus can be recognized by the loss of surrounding fat tissue and T2 hyperintensity within the plexus fibers [41,42] (Figure 12).

Magnetic resonance angiography (MRA) can be used particularly when symptoms suggest arterial or venous compression in addition to the neurogenic component. MRA offers a noninvasive method to assess both static anatomical abnormalities and dynamic vascular changes during provocative maneuvers [1,37]. The main advantages of MRA include superior soft tissue contrast, the ability to assess neurovascular structures simultaneously, and the absence of ionizing radiation, which is particularly beneficial in younger patients [41].

**Figure 13:**
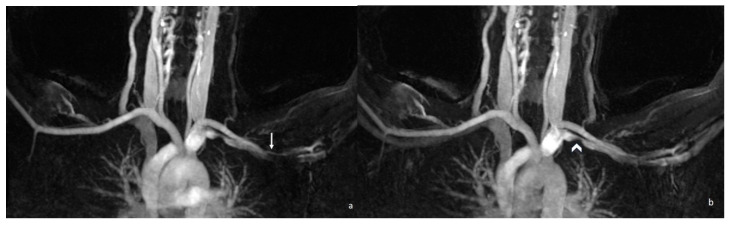
TWIST MR angiography images obtained with both arms elevated demonstrate narrowing of the left subclavian artery (**a**) (arrow) and left subclavian vein (**b**) (arrowhead). TWIST = time-resolved MR angiography with interleaved stochastic trajectories.

Contrast-enhanced MRA is the standard for vascular evaluation. It provides high spatial resolution for detecting stenosis, aneurysm, or thrombosis. This technique requires careful bolus timing to differentiate arterial and venous phases [1]. Time-resolved MRA techniques such as TWIST (Time-Resolved MR Angiography with Interleaved Stochastic Trajectories) enable acquisition of high-temporal-resolution 3D datasets with lower contrast doses. They allow for separation of arterial and venous phases, even in patients with variable hemodynamics. Time-resolved MRA techniques are particularly valuable for capturing transient compression phenomena during provocative maneuvers [44,45,46] (Figure 13).

**Figure 14:**
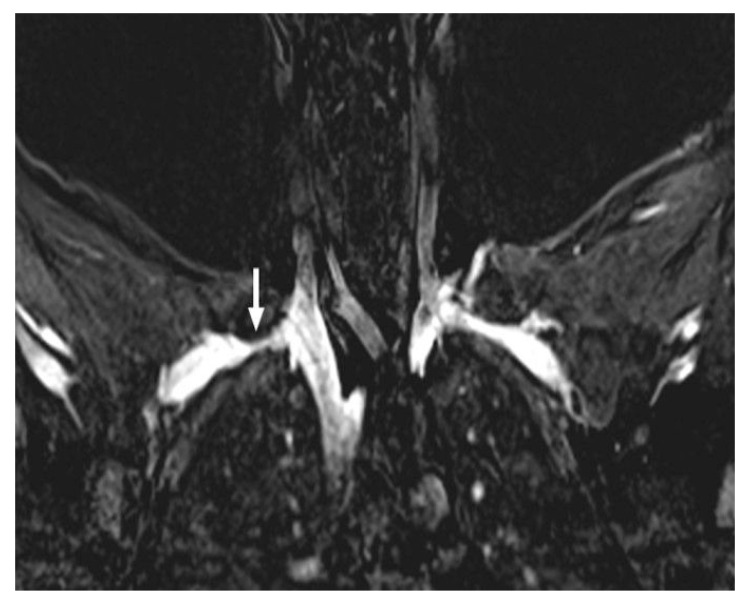
3D VIBE image obtained with both arms elevated demonstrates narrowing of the right subclavian vein (arrow). 3D VIBE = three-dimensional volumetric interpolated breath-hold examination.

3D VIBE (Volumetric Interpolated Breath-Hold Examination) is a high-resolution isotropic imaging technique suitable for multiplanar reconstruction. It provides excellent depiction of both arterial lumen and venous structures. It is helpful for demonstrating extrinsic compressive masses adjacent to the vascular bundle [47] (Figure 14). In cases of combined neurovascular involvement, MRA can also delineate mass lesions or anomalous musculature like hypertrophied scalene muscles, pectoralis minor syndrome that exerts extrinsic compression on both nerves and vessels, allowing a comprehensive assessment in a single examination [2,41]. The MRI protocol for TOS is summarized in Table 3.

There are some limitations and challenges with the use of MRA. Motion artifacts can occur due to long acquisition times, especially in patients with pain or difficulty maintaining provocative positions. Bolus timing in CE-MRA can be challenging and improper timing may lead to overlap of arterial and venous phases. Limited bore size of the MRI scanner can restrict arm positioning [41].

### 5.4. Ultrasonography

Ultrasonography (US) is a noninvasive, radiation-free, inexpensive, and portable imaging modality that can be used as an initial diagnostic tool in patients with suspected TOS [28]. Its main advantages include the ability to perform imaging in various patient positions (sitting, standing, or supine) and during provocative maneuvers in addition to the neutral position [1,48]. However, limited acoustic windows—particularly in the costoclavicular region—represent a major limitation. Operator dependency and technical challenges in obese patients are additional drawbacks [2]. Since US cannot adequately visualize the entire thoracic outlet or the lung apices, it may fail to detect alternative pathologies such as superior sulcus tumors; therefore, it should not be used as a standalone modality for the diagnosis of TOS [1]. Its diagnostic value increases in patients with positive clinical findings when CT and MRI results are negative [49].

**Figure 15:**
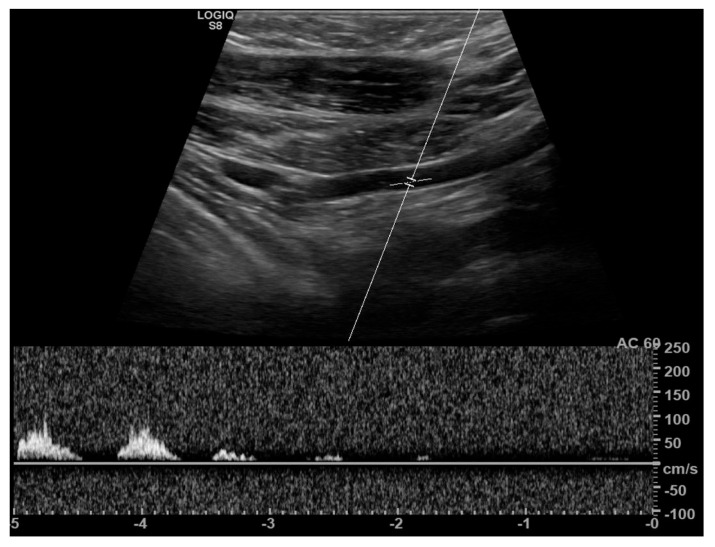
Spectral Doppler ultrasound demonstrates a monophasic waveform with progressively decreasing flow velocity in the left subclavian artery at the retropectoralis minor space.

Real-time duplex ultrasonography, combining B-mode and Doppler imaging, is performed both in the neutral position and during provocative maneuvers such as the Adson, Eden, and 90-degree Wright tests [10]. An increase in arterial flow velocity due to turbulence or complete signal loss during these maneuvers is considered suggestive of TOS [50,51]. (Figure 15). However, these findings reflect indirect evidence of proximal arterial stenosis and do not precisely identify the level of compression. B-mode imaging may also demonstrate poststenotic dilatation, aneurysm formation, and vascular deviation [1].

US has an established role in the diagnosis of venous TOS, particularly in detecting upper extremity venous thrombosis. In cases of thrombosis, B-mode imaging shows increased intraluminal echogenicity, loss of color Doppler signal, and venous noncompressibility [2,52,53].

The role of US in evaluating neural structures is limited and highly operator dependent [54]. Fouasson-Chailloux et al. reported that, despite a significant association between NTOS and vascular compression, duplex ultrasonography has limited diagnostic value due to its low sensitivity and specificity and cannot reliably establish the diagnosis of NTOS [55]. Visualization of the brachial plexus is challenging due to the complex regional anatomy and acoustic shadowing from adjacent osseous structures. In contrast, a prospective study has described a potential ultrasonographic finding in TOS. The so-called “wedged sickle sign” refers to an echogenic fibromuscular structure at the medial border of the middle scalene muscle, associated with thickening and hypoechogenicity of the lower trunk of the brachial plexus [56]. However, this finding has been reported in limited studies and requires further validation before it can be considered clinically reliable.

### 5.5. Digital Subtraction Angiography

**Figure 16:**
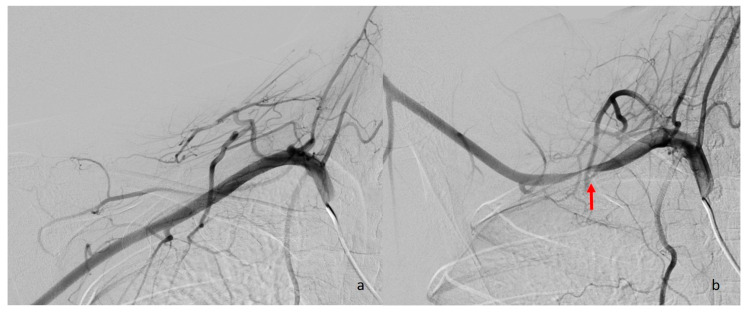
Arterial-phase DSA images show that the right subclavian artery, normal in caliber in the neutral position (**a**), becomes compressed during arm hyperabduction (**b**) (red arrow).

**Figure 17:**
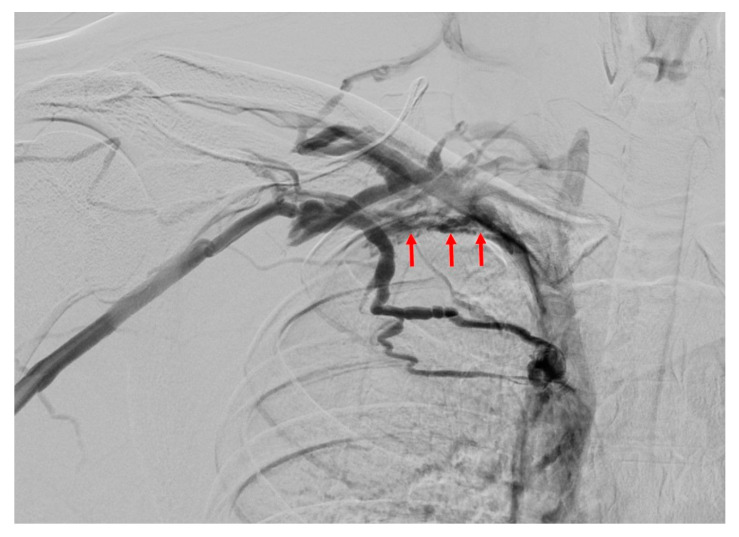
Venous-phase DSA examination reveals thinning and wall irregularities (red arrows) in the left subclavian vein due to chronic thrombosis, along with extensive venous collaterals in the surrounding area.

While digital subtraction angiography (DSA) can demonstrate vascular compression in both arteries and veins, it does not provide information about the underlying anatomical cause of the compression (Figure 16). In recent years, less invasive imaging modalities such as CTA and MRA have largely replaced DSA for diagnostic purposes, as they allow for direct visualization of the structures responsible for compression [2]. However, DSA still plays a role in guiding interventional procedures [10]. In cases of arterial or venous TOS with acute symptoms and confirmed thrombosis, DSA is typically used to facilitate thrombolytic agent infusion or mechanical thrombectomy prior to definitive surgical intervention [2,57,58] (Figure 17).

The imaging modalities and typical radiologic findings observed with each imaging modality and their relevance to TOS subtypes are summarized in Table 4.

## 6. Conclusions

TOS represents a complex and heterogeneous clinical entity in which accurate diagnosis relies on a thorough understanding of thoracic outlet anatomy, potential etiological factors, and the dynamic nature of neurovascular compression. Imaging plays a central role in the diagnostic evaluation of TOS by confirming neurovascular compression, identifying the affected structures, localizing the level of compromise, and revealing the underlying anatomical or pathological abnormalities. Equally important, imaging allows exclusion of alternative conditions that may mimic TOS and significantly influence patient management.

No single imaging modality alone is sufficient for the comprehensive evaluation of all TOS subtypes. Instead, an individualized, multimodality imaging strategy—guided by clinical presentation and suspected subtype—is required. Importantly, positional vascular narrowing may be observed in asymptomatic individuals; therefore, imaging findings should never be interpreted in isolation. Close clinical correlation is essential to avoid overdiagnosis and unnecessary surgical intervention. A standardized, imaging-informed, multidisciplinary approach is crucial for optimizing diagnostic accuracy, guiding appropriate treatment, and improving clinical outcomes in patients with thoracic outlet syndrome.

## Figures and Tables

**Table 1:** Etiology of TOS.

**Bone Abnormalities**	
*Congenital*	*Acquired*
Cervical rib Elongated C7 transverse process Broad first rib Enlarged scalene tubercle Hypoplastic first rib that articulates with second rib	Exostoses or tumors of first rib or clavicle Hypertrophic callus formation of first rib or clavicle
**Soft tissue abnormalities**	
*Congenital*	*Acquired*
Fibrous bands Hypertrophy or abnormal insertions of the anterior scalene muscle Distal division of a single-origin anterior and middle scalene muscle Brachial plexus passing between fibers of the scalene muscles Interdigitation of scalene muscles Insertion of the middle scalene muscle onto the first rib with a broad base Accessory scalenus minimus muscle Hypertrophy of the subclavius muscle Accessory subclavius posticus muscle Variations in dorsal scapular artery origin	Posttraumatic fibrous scarring (direct flexion- extension trauma to the neck, repetitive microtraumas—often seen in sports, occupational tasks, and other activities that involve frequent overhead arm movements or heavy lifting) Postoperative fibrous scarring Hyperactive pectoralis minor muscle (due to chronic muscle contraction and fibrosis)
**Predisposing postural or morphologic factors**	
Droopy shoulder syndrome	
Poor posture and weak shoulder muscles	
**Pathologies that mimic TOS symptoms**	
Superior sulcus tumor of the lung	
Tumors located in the supraclavicular region (lipomas and neurogenic tumors)	
Large vessel vasculitis (Takayasu arteritis)	
Advanced atherosclerosis	

TOS = thoracic outlet.

**Table 2:** CT protocol for TOS.

Place the intravenous line in the **contralateral arm** to avoid venous streak artifacts. Inject **90 mL iodinated contrast** via the **antecubital vein** at **4 mL/s.** Start the scan **15–20 s after injection.** Timing may be performed using the **bolus-tracking method.** If **venous TOS** is suspected, add a **delayed scan at 90 s.**
Perform the examination in **two positions**: Scan with the **symptomatic arm in neutral position (adduction)** while the **contralateral** ** arm is abducted** to reduce streak artifacts. Scan with the **symptomatic arm in abduction** while the **contralateral arm is adducted**.
Reconstructions with **thin slice thickness (1–2 mm):** **2D reformatted** images; Axial, coronal, sagittal *; **3D reformatted** images **.

* Sagittal reformatted images allow more accurate assessment of the location and degree of arterial compression. ** Three-dimensional volumetric reconstructions better demonstrate the relationship between osseous and vascular structures and facilitate the identification of poststenotic dilatation. TOS = thoracic outlet; 2D = two-dimensional; 3D = three-dimensional.

**Table 3:** MR imaging protocol for TOS.

**Coil** Phased-array head/neck or body coil
**Sequences** *In neutral position:* 3D coronal T2-weighted TSE; Sagittal T2-weighted TSE; Sagittal T1-weighted TSE; Sagittal STIR; Axial T2-weighted GRE; Coronal T2-weighted STIR; Coronal T1-weighted TSE; Axial T2-weighted TSE; Axial STIR; Sagittal oblique T2-weighted imaging. *In hyperabduction:* Sagittal oblique T2-weighted imaging; Time-resolved contrast-enhanced MR angiography (e.g., TWIST) or 3D VIBE MR angiography.
**Contrast material** Gadolinium-based contrast agent (0.1 mmol/kg), 1–1.5 mL/s

TOS = thoracic outlet; 3D = three-dimensional; TSE = turbo spin-echo; STIR = short tau inversion recovery; GRE = gradient-echo; TWIST = time-resolved MR angiography with interleaved stochastic trajectories; 3D VIBE = Volumetric Interpolated Breath-Hold Examination.

**Table 4:** Imaging modalities used in the diagnosis of TOS and typical imaging findings according to subtypes.

Imaging Modality	Main Advantages	Limitations	Best Use in TOS Subtype	Typical Findings
**Radiography**	Non-invasive, inexpensive, low radiation exposure	Only bone abnormalities and lesions can be visualized	First-line in all three types of TOS	Cervical ribs, elongated C7 transverse process, congenital or acquired deformities of the first rib and clavicle, focal bone lesions
**CTA**	Excellent spatial resolution, detailed evaluation of bone and vessels, detects aneurysm/stenosis	Radiation exposure, iodinated contrast required	Arterial TOS, osseous abnormalities (cervical rib and first rib anomalies)	Arterial stenosis or aneurysm, bony abnormalities (cervical rib, first rib anomaly), positional compression (dynamic CTA)
**CTV**	High sensitivity for venous thrombosis or stenosis	Radiation exposure, iodinated contrast required	Venous TOS, especially effort thrombosis	Subclavian vein stenosis or thrombosis, collateral venous formation
**MRI**	Excellent soft tissue contrast, evaluates brachial plexus and surrounding muscles, direct visualization of nerve compression and signal changes	Longer acquisition time, motion sensitivity	Neurogenic TOS, soft tissue abnormalities	Brachial plexus signal abnormalities, soft tissue abnormalities (fibromuscular bands, hypertrophic scalene, etc.), muscle or nerve lesions
**MRA**	Non-ionizing and good vascular visualization	Lower spatial resolution than CTA	Alternative to CTA in arterial or venous TOS	Vascular stenosis, thrombosis, aneurysm, positional changes (dynamic MRA)
**US**	Non-invasive, inexpensive, radiation-free, dynamic evaluation possible, real-time vascular flow assessment	Operator-dependent, limited acoustic windows limited evaluation of deep structures	First-line in venous and arterial TOS; dynamic compression assessment	Increased intraluminal echogenicity, loss of color Doppler signal, and venous noncompressibility in venous thrombosis, flow changes on positional maneuvers, arterial peak velocity changes
**DSA**	Gold standard for vascular anatomy, allows real-time intervention (thrombolytic agent infusion or mechanical thrombectomy)	Invasive, contrast and radiation exposure, inability to identify anatomical cause of the compression	Preoperative evaluation of arterial TOS, interventions for arterial or venous TOS	Arterial and venous stenosis or occlusion, aneurysm, collateral vessels, positional compression

TOS = thoracic outlet; US = ultrasonography; CTA = computed tomographic angiography; CTV = computed tomographic venography; MRI = magnetic resonance imaging; MRA = magnetic resonance angiography; DSA = digital subtraction angiography.

## Data Availability

The datasets generated and analyzed during the current study are available from the corresponding author on reasonable request.

## References

[1] Demondion X., Herbinet P., Van Sint Jan S., Boutry N., Chantelot C., Cotten A. (2006). Imaging assessment of thoracic outlet syndrome. Radiographics.

[2] Raptis C.A., Sridhar S., Thompson R.W., Fowler K.J., Bhalla S. (2016). Imaging of the patient with thoracic outlet syndrome. Radiographics.

[3] Daley P., Pomares G., Gross R., Menu P., Dauty M., Fouasson-Chailloux A. (2022). Use of electroneuromyography in the diagnosis of neurogenic thoracic outlet syndrome: A systematic review and meta-analysis. J. Clin. Med..

[4] Khalilzadeh O., Glover M., Torriani M., Gupta R. (2021). Imaging assessment of thoracic outlet syndrome. Thorac. Surg. Clin..

[5] de Kleijn R.J.C.M.F., Schropp L., Westerink J., van Hattum E.S., Petri B.J., de Borst G.J. (2023). Functional outcome of arterial thoracic outlet syndrome treatment. Front. Surg..

[6] Illig K.A., Gober L. (2022). Optimal management of upper extremity deep vein thrombosis: Is venous thoracic outlet syndrome underrecognized?. J. Vasc. Surg. Venous Lymphat. Disord..

[7] Karaolanis G., Antonopoulos C.N., Koutsias S.G., Giosdekos A., Metaxas E.K., Tzimas P., de Borst G.J., Geroulakos G. (2021). A systematic review and meta-analysis for the management of Paget–Schroetter syndrome. J. Vasc. Surg. Venous Lymphat. Disord..

[8] Illig K.A., Donahue D., Duncan A., Freischlag J., Gelabert H., Johansen K., Jordan S., Sanders R., Thompson R. (2016). Reporting standards of the Society for Vascular Surgery for thoracic outlet syndrome. J. Vasc. Surg..

[9] Illig K.A., Rodriguez-Zoppi E., Bland T., Muftah M., Jospitre E. (2021). The incidence of thoracic outlet syndrome. Ann. Vasc. Surg..

[10] Moriarty J.M., Bandyk D.F., Broderick D.F., Cornelius R.S., Dill K.E., Francois C.J., Gerhard-Herman M.D., Ginsburg M.E., Hanley M., Kalva S.P. (2015). ACR appropriateness criteria imaging in the diagnosis of thoracic outlet syndrome. J. Am. Coll. Radiol..

[11] Altuwaijri T.A. (2022). Comparison of duplex ultrasound and hemodynamic assessment with computed tomography angiography in patients with arterial thoracic outlet syndrome. Medicine.

[12] Remy-Jardin M., Remy J., Masson P., Bonnel F., Debatselier P., Vinckier L., Duhamel A. (2000). Helical CT angiography of thoracic outlet syndrome: Functional anatomy. AJR Am. J. Roentgenol..

[13] Demondion X., Bacqueville E., Paul C., Duquesnoy B., Hachulla E., Cotten A. (2003). Thoracic outlet: Assessment with MR imaging in asymptomatic and symptomatic populations. Radiology.

[14] Demondion X., Boutry N., Drizenko A., Paul C., Francke J.P., Cotten A. (2000). Thoracic outlet: Anatomic correlation with MR imaging. AJR Am. J. Roentgenol..

[15] Chang K.Z., Likes K., Davis K., Demos J., Freischlag J.A. (2013). The significance of cervical ribs in thoracic outlet syndrome. J. Vasc. Surg..

[16] Al Subhi M., Al Ajmi E., Al Lawati A., Al Aswami H., Chan M.F., Sirasanagandla S.R. (2022). Prevalence of cervical ribs and elongated transverse processes in Omani population: A computed tomography-based study. Surg. Radiol. Anat..

[17] Jiang D., Weiss R., Lind B., Morcos O., Lee C.J. (2024). Predisposing anatomy for thoracic outlet syndrome and functional outcomes after supraclavicular thoracic outlet decompression in athletes. Vasc. Spec. Int..

[18] Ajalat M.J., Pantoja J.L., Ulloa J.G., Cheng M.J., Patel R.P., Chun T.T., Gelabert H.A. (2022). A single institution 30-year review of abnormal first rib resection for thoracic outlet syndrome. Ann. Vasc. Surg..

[19] Tafti A.A., Salmani M. (2025). Operative treatment of clavicle nonunion complicated with acute thoracic outlet syndrome: A case report and review of literature. JSES Rev. Rep. Tech..

[20] Kraft J., Contrucci A.L. (2023). Rib pseudoarthrosis with thoracic outlet syndrome in pediatric gymnast: A case report. J. Med. Case Rep..

[21] Buero A., Olivera Lopez S., Pereyra C., David M., Pankl L.G., Samudio M., Méndez J., Lyons G.A., Chimondeguy D.J. (2025). Thoracic outlet syndrome secondary to a cavernous hemangioma of the first rib. Medicina.

[22] Chen Y., Deng K., Zhao C., Xiao W., Tang Z. (2024). Resection of the entire first rib for giant osteochondroma by trans manubrial approach: A case report and review of the literature. J. Cardiothorac. Surg..

[23] Hamouri S., AlQudah M., Al-Zoubi N., Al Gargaz W., Jarboa’ H., Hecker E. (2021). Rib osteoblastoma as a cause of neurogenic thoracic outlet syndrome: A case report. Am. J. Case Rep..

[24] Kitsis C.K., Marino A.J., Krikler S.J., Birch R. (2003). Late complications following clavicular fractures and their operative management. Injury.

[25] Ha J., Lee S., Baek J., Ryou K.S., Park T.J., Kim S.H. (2023). Neurogenic thoracic outlet syndrome induced by subclavius muscle hypertrophy: A case report. Nerve.

[26] Al Redouan A., Benes M., Abbaspour E., Kunc V., Kachlik D. (2023). Prevalence and anatomy of the anomalous subclavius posticus muscle and its clinical implications with emphasis in neurogenic thoracic outlet syndrome: Scoping review and meta-analysis. Ann. Anat..

[27] Rizzo S., Talei Franzesi C., Cara A., Cassina E.M., Libretti L., Pirondini E., Raveglia F., Tuoro A., Vaquer S., Degiovanni S. (2024). Diagnostic and therapeutic approach to thoracic outlet syndrome. Tomography.

[28] Ahmed A.S., Lafosse T., Graf A.R., Karzon A.L., Gottschalk M.B., Wagner E.R. (2023). Modern treatment of neurogenic thoracic outlet syndrome: Pathoanatomy, diagnosis, and arthroscopic surgical technique. J. Hand Surg. Glob. Online.

[29] Verenna A.A., Alexandru D., Karimi A., Brown J.M., Bove G.M., Daly F.J., Pastore A.M., Pearson H.E., Barbe M.F. (2016). Dorsal scapular artery variations and relationship to the brachial plexus, and a related thoracic outlet syndrome case. J. Brachial Plex. Peripher. Nerve Inj..

[30] Hanna A., Bodden L.O., Siebiger G.R.L. (2018). Neurogenic thoracic outlet syndrome caused by vascular compression of the brachial plexus: A report of two cases. J. Brachial Plex. Peripher. Nerve Inj..

[31] Lai K.C., Ho H.C. (2023). Origin variations and brachial plexus relationship of the dorsal scapular artery. Sci. Rep..

[32] Lokman B., Aymane A., Yachaoui S., El Oumri A.A. (2024). Droopy shoulder syndrome: A gateway to thoracic outlet syndrome. Cureus.

[33] Çiflik K.B., Özdemir Çiflik B. (2025). Thoracic outlet syndrome induced by extrathoracic giant lipoma: First case in the literature due to the atypical location. J. Cardiothorac. Surg..

[34] Kawano K., Hara Y., Hoshikawa S., Tajiri Y. (2023). Thoracic outlet syndrome caused by a primary tumour in the brachial plexus. J. Hand Surg. Asian Pac. Vol..

[35] Almourgi M.A. (2024). A rare case of adult cervicothoracic cystic lymphangioma presenting as neurogenic thoracic outlet syndrome. Cureus.

[36] Remy-Jardin M., Remy J., Masson P., Bonnel F., Debatselier P., Vinckier L., Duhamel A. (2000). CT angiography of thoracic outlet syndrome: Evaluation of imaging protocols for the detection of arterial stenosis. J. Comput. Assist. Tomogr..

[37] Ghouri M.A., Gupta N., Bhat A.P., Thimmappa N.D., Saboo S.S., Khandelwal A., Nagpal P. (2019). CT and MR imaging of the upper extremity vasculature: Pearls, pitfalls, and challenges. Cardiovasc. Diagn. Ther..

[38] Hasanadka R., Towne J.B., Seabrook G.R., Brown K.R., Lewis B.D., Foley W.D. (2007). Computed tomography angiography to evaluate thoracic outlet neurovascular compression. Vasc. Endovasc. Surg..

[39] Schropp L., Vonken E.J., Smits M.L.J., de Graaf E.K.L., Gillebaard S.E., Bots M.L., van Hattum E.S., Petri B.J., de Borst G.J. (2025). Quantitative computed tomography and magnetic resonance assessment of vascular compression in the thoracic outlet. Eur. J. Vasc. Endovasc. Surg..

[40] Szaro P., Suresh R., Molokwu B., Sibala D.R., Mendiratta D., Chu A., McGrath A. (2023). Magnetic resonance imaging for diagnosis of suspected neurogenic thoracic outlet syndrome: A systematic scoping review. Front. Physiol..

[41] Gilcrease-Garcia B.M., Deshmukh S.D., Parsons M.S. (2020). Anatomy, imaging, and pathologic conditions of the brachial plexus. Radiographics.

[42] Chhabra A., Thawait G.K., Soldatos T., Thakkar R.S., Del Grande F., Chalian M., Carrino J.A. (2013). High-resolution 3T MR neurography of the brachial plexus and its branches, with emphasis on 3D imaging. AJNR Am. J. Neuroradiol..

[43] Cejas C., Rollán C., Michelin G., Nogués M. (2016). High resolution neurography of the brachial plexus by 3 Tesla magnetic resonance imaging. Radiologia.

[44] Vogt F.M., Theysohn J.M., Michna D., Hunold P., Neudorf U., Kinner S., Barkhausen J., Quick H.H. (2013). Contrast-enhanced time-resolved 4D MRA of congenital heart and vessel anomalies: Image quality and diagnostic value compared with 3D MRA. Eur. Radiol..

[45] Kim C.Y., Merkle E.M. (2008). Time-resolved MR angiography of the central veins of the chest. AJR Am. J. Roentgenol..

[46] Lauenstein T., Umutlu L., Fischer A., Quinsten A., Kloeters C., Kinner S. Time-resolved MR angiography with interleaved stochastic trajectories (TWIST) for the diagnosis of thoracic outlet syndrome. Proceedings of the Radiological Society of North America (RSNA) 97th Scientific Assembly and Annual Meeting.

[47] Zhang T., Xu Z., Chen J., Liu Z., Wang T., Hu Y., Shen L., Xue F. (2019). A novel approach for imaging of thoracic outlet syndrome using CE-MRA, T2-STIR-SPACE, and VIBE. Med. Sci. Monit..

[48] Zurkiya O., Ganguli S., Kalva S.P., Chung J.H., Shah L.M., Majdalany B.S., Bykowski J., Carter B.W., Chandra A., Collins J.D. (2020). ACR appropriateness criteria^®^ thoracic outlet syndrome. J. Am. Coll. Radiol..

[49] Demondion X., Vidal C., Herbinet P., Gautier C., Duquesnoy B., Cotten A. (2006). Ultrasonographic assessment of arterial cross-sectional area in the thoracic outlet on postural maneuvers measured with power Doppler ultrasonography. J. Ultrasound Med..

[50] Bishop L., Bartlett M. (2021). Doppler waveform analysis during provocative manoeuvres in the assessment for arterial thoracic outlet syndrome results in high false-positive rates: A cross-sectional study. JRSM Cardiovasc. Dis..

[51] Simpson T., Safir S., Radulovic M., Hines G. (2025). Thoracic outlet syndrome: A comprehensive review. Cardiol. Rev..

[52] Taha K., Breslin T., Moriarty J.M., Ali S., Louw B. (2022). Diagnosing Paget–Schroetter syndrome using point of care ultrasound (POCUS). POCUS J..

[53] Khan O., Marmaro A., Cohen D.A. (2021). A review of upper extremity deep vein thrombosis. Postgrad. Med..

[54] Demondion X., Herbinet P., Boutry N., Fontaine C., Francke J.P., Cotten A. (2003). Sonographic mapping of the normal brachial plexus. AJNR Am. J. Neuroradiol..

[55] Fouasson-Chailloux A., Menu P., Daley P., Gautier G., Gadbled G., Abraham P., Dauty M. (2021). Subclavian vessel compression assessed by duplex scanning in patients with neurogenic thoracic outlet syndrome and no vascular signs. Diagnostics.

[56] Arányi Z., Csillik A., Böhm J., Schelle T. (2016). Ultrasonographic identification of fibromuscular bands associated with neurogenic thoracic outlet syndrome: The “wedge-sickle” sign. Ultrasound Med. Biol..

[57] Elsabbagh M.A., El Sayed Salem M., Mabrouk M.K., El-Emam A.A., Gaweesh A., Tiwari A. (2025). Early results of interventions in patients with venous thoracic outlet syndrome. Ann. Vasc. Surg..

[58] Sen I., DeMartino R., Bjarnason H., Neisen M., Kalra M. (2023). Results of a flexible patient-centered approach to the timing of thoracic outlet decompression in Paget–Schroetter syndrome. Ann. Vasc. Surg..

